# Whey Protein Concentrate/Isolate Biofunctional Films Modified with Melanin from Watermelon (*Citrullus lanatus*) Seeds

**DOI:** 10.3390/ma13173876

**Published:** 2020-09-02

**Authors:** Łukasz Łopusiewicz, Emilia Drozłowska, Paulina Trocer, Mateusz Kostek, Mariusz Śliwiński, Marta H. F. Henriques, Artur Bartkowiak, Peter Sobolewski

**Affiliations:** 1 Center of Bioimmobilisation and Innovative Packaging Materials, Faculty of Food Sciences and Fisheries, West Pomeranian University of Technology Szczecin, Janickiego 35, 71-270 Szczecin, Poland; emilia_drozlowska@zut.edu.pl (E.D.); p.trocer@gmail.com (P.T.); mkosa9406@gmail.com (M.K.); Artur-Bartkowiak@zut.edu.pl (A.B.); 2 Dairy Industry Innovation Institute Ltd., Kormoranów 1, 11-700 Mrągowo, Poland; mariusz.sliwinski@iipm.pl; 3 Polytechnic Institute of Coimbra, College of Agriculture, Bencanta, PT-3045-601 Coimbra, Portugal; mhenriques@esac.pt; 4 CERNAS—Research Center for Natural Resources, Environment and Society, Polytechnic Institute of Coimbra, Bencanta, PT-3045-601 Coimbra, Portugal; 5 Department of Polymer and Biomaterials Science, Faculty of Chemical Technology and Engineering, West Pomeranian University of Technology Szczecin 45 Piastów Ave, 70-311 Szczecin, Poland; piotr.sobolewski@zut.edu.pl

**Keywords:** melanin, watermelon seeds, whey protein, bioactive films, plant residues

## Abstract

Valorization of food industry waste and plant residues represents an attractive path towards obtaining biodegradable materials and achieving “zero waste” goals. Here, melanin was isolated from watermelon (*Citrullus lanatus*) seeds and used as a modifier for whey protein concentrate and isolate films (WPC and WPI) at two concentrations (0.1% and 0.5%). The modification with melanin enhanced the ultraviolet (UV) blocking, water vapor barrier, swelling, and mechanical properties of the WPC/WPI films, in addition to affecting the apparent color. The modified WPC/WPI films also exhibited high antioxidant activity, but no cytotoxicity. Overall, the effects were melanin concentration-dependent. Thus, melanin from watermelon seeds can be used as a functional modifier to develop bioactive biopolymer films with good potential to be exploited in food packaging and biomedical applications.

## 1. Introduction

The food market represents a large part of the global economy and is growing every year. Hand-in-hand, this economic sector is now also responsible for approx. 1.3 billion tons of waste per annum [1]. This waste, from fruit, vegetable, and food, includes waste generated during all aspects of food production: cleaning, processing, cooking, and packaging. However, some of these waste products and/or by-products can be important sources of bioactive compounds, such as phenolic compounds, dietary fiber, polysaccharides, vitamins, carotenoids, pigments, and oils [2]. These compounds can be potentially used in the development of novel food products (food additives and functional foods) or food packaging materials. This is an attractive path towards waste valorization in line with current market trends connected with “zero waste” goals and the so-called circular economy [3,4]. Therefore, continuing research into both the characterization and utilization of compounds obtained from food-industry waste/by-products is important, because it may offer a path towards improved sustainability of the food industry. This could significantly mitigate environmental problems associated with this industry, as well as have a positive impact from the point of view of climate change [1,2,5].

Watermelon (*Citrullus lanatus*, clade: Rosids, order: Cucurbitales, family: *Cucurbitaceae*) is a very popular fruit, with the flesh both consumed, as well as processed into juice and juice concentrates, due to water content approaching 92% of total weight. However, watermelon seeds, which constitute about 1 to 4% of total fruit weight, are not routinely eaten with the pulp [6,7,8,9]. At the same time, these seeds do have economic value, particularly in countries where cultivation is increasing. They can be used to prepare snacks or be milled into flour and used in sauces. Watermelon seeds are reported to be a rich source of proteins, vitamins B and E, minerals (such as magnesium, potassium, phosphorous, sodium, iron, zinc, manganese and copper), polyunsaturated fatty acids such as omega-6 (linoleic acid), and monounsaturated fatty acids, such as omega-9 (oleic acid). They also consist of saturated fatty acids, such as palmitic acid and stearic acid, and were found to be rich in γ-sitosterol, β-sitosterol, and lupeol [6,7,8,9]. Further, they are a promising source of useful compounds with potential biofunctional properties such as polyphenols, saponins, alkaloids and flavonoids [10,11]. However, despite these applications, watermelon seeds are still typically discarded, with only the fruit being eaten [10,12].

Biodegradable edible films are defined as a thin layer of material, that can be consumed. They are typically used to extend the shelf life and/or to improve the quality of foods. For example, they can be used to act as barriers to mass transfer, carriers of specific ingredients, or for the improvement of mechanical/handling characteristics of the product [13,14,15]. Growing consumer demand for high-quality foods, along with increasing environmental concern regarding the disposal of non-renewable food packaging materials, has led to a great deal of interest in the development of novel, biodegradable edible films/coatings [1,15,16]. However, such films, which are typically composed of biopolymers, can be also used in biomedical applications i.e., as wound dressings [17]. Further, the functional properties of such biopolymer films can be improved by adding different biofunctional compounds (e.g., antioxidant and/or antimicrobial properties). In this fashion, one can obtain biodegradable, bioactive materials with properties suitable for a range of diverse applications, while reducing the use of synthetic chemical additives that may have negative on human health or the environment [1,15,16].

At present, the packaging industry is dominated by synthetic polymers (plastics), because they are very cheap and possess good mechanical and physical properties. The annual plastic production is estimated to be approx. 300 million tons, of which 40% is used in packaging. However, this wide use of synthetic packaging materials has caused serious concerns, due to their high environmental impact [1,18]. Synthetic packaging polymers are petroleum-based and thus non-renewable, while at the same time being typically non-biodegradable. As a result, packaging accounts for large amounts of waste materials and pollution in the environment [1,13,19]. As a result, there is a pressing need to develop new, more eco-friendly packaging materials. In this context, biopolymers are very promising, because, compared to petroleum-based synthetic plastics, they are derived from a biological origin, making them renewable, biodegradable, and non-toxic or biocompatible [1,14]. A wide range of carbohydrates, proteins, and lipids—all derived from renewable sources—are being investigated as biodegradable alternatives, to improve sustainability and recyclability [1,13,20,21]. In particular, protein-based films are promising, due to better mechanical attributes, barrier characteristics, and nutritional-promoting properties, as compared to polysaccharide and lipid-based materials [20,22]. Gradually, bio-sourced materials are likely to replace the commonly utilized petroleum-based polymers, as environmental and sustainability externalities become increasingly accounted for in their cost [23].

Among protein-based edible films, whey protein (WP) films have received increased interest, because they possess interesting sensorial, optical, and mechanical barrier properties [24,25]. Whey is a protein-rich, major by-product of the cheese manufacturing industry [1,24,26]. In fact, this industry generates large volumes of fluid whey, that need to be properly disposed of, in order to avoid potential environmental problems [25]. Thus, whey protein-based edible films and coatings are not only value-added products, but also offer a potential solution to the disposal problem [1]. Heat-denatured whey proteins, with the addition of a plasticizer, yield transparent, bland, and flexible films with very good resistance to oxygen, aroma, and lipid transfer at low humidity [15,23]. However, the hydrophilic nature of the proteins enables interactions with water, which leads to a reduction in the moisture barrier properties [25,27]. In addition to applications in edible films, whey protein concentrates and isolates (WPC, WPI) also have the potential to be used in the biomedical field, for example forming hydrogels as bioactive carriers or by leveraging their antioxidative properties [28,29,30,31].

Melanins are black and brown biopigments, consisting of high molecular weight heterogeneous polymers derived from the oxidation of monophenols and the subsequent polymerization of intermediate *o*-diphenols and their resulting quinones [32]. The molecular structure of melanins includes multiple different reactive functional groups (−OH, −NH, and −COOH) [21]. They can be obtained and have been characterized by a variety of natural sources, including animals, plants, bacteria, and fungi [10,11,33,34]. Importantly, melanins are multifunctional and biologically-active, natural macromolecules and can be characterized as antioxidant, radioprotective, thermo-regulative, chemoprotective, antitumor, antiviral, antimicrobial, immunostimulating and/or anti-inflammatory [10,32,34,35,36]. Potentially, melanins could be used to impart some of these important attributes to polymers. In the case of biopolymers, this could enhance performance, as well as sustainability credentials. Further, melanins could enable a wide range of applications, for example by facilitating cross-linking during polymerization, providing antioxidant or antimicrobial activity, altering light scattering ability, or improving other biological properties of the polymers [18,19,37]. Importantly, melanins, like biopolymers, are obtained from renewable resources and are non-toxic; these two features make their use “greener” than many existing commercial additives [35]. Importantly, large-scale production of melanins by microorganisms digesting food waste, as well as by sustainable extraction from natural plant-residues (e.g., watermelon seeds) have been demonstrated [10,11,34]. In fact, melanin from watermelon seeds has been shown to have antioxidant and UV-barrier properties [10]. However, compared to their potential, the use of melanins remains under-explored. The relatively few examples typically involve their blending/use with polymers (as chemical modifiers or nanofillers) to modify films and coatings, for example: gelatin [38,39], poly(lactic acid) [18], alginate [21], agar [19], carrageenan [13], cellulose [22], chitosan [14], poly(vinyl alcohol) [40], polypropylene/poly(butylene adipate-co-terephthalate) [37], polyhydroxybutyrate [41] and, ethylene-vinyl acetate copolymer [35].

In this study, our aim was to investigate the effect of adding melanin obtained from watermelon seed on the properties of whey protein concentrate/isolate (WPC/WPI) films. To the best of our knowledge, no reports have been published on the modification of WPC or WPI films with natural melanin to improve the functionality of the materials. We used UV-Vis and IR spectroscopy to examine the chemical composition of films after melanin addition. Additionally, we also assessed the influence of melanin on the color, hydrodynamic, and optical properties of the films. Finally, in order to evaluate the potential (bio) functionality of the obtained materials, we evaluated their mechanical, barrier, and antioxidant properties and screened for any potential cytotoxicity in vitro.

## 2. Materials and Methods

### 2.1. Materials and Reagents

Whey protein concentrate (WPC, 85% protein content) and whey protein isolate (WPI, 90% protein content) manufactured from sweet cheese whey using cross-flow membrane filtration were purchased from Volac International Ltd. (Hertfordshire, UK). Calcium chloride, hydrogen peroxide, disodium phosphate, monosodium phosphate, 2,2-diphenyl-1-picrylhydrazyl (DPPH), 2,2′-azino-bis(3-ethylbenzothiazoline-6-sulfonic acid) (ABTS), potassium persulphate, potassium ferricyanide, trichloroacetic acid, ferric chloride, iron sulphate, tris(hydroxymethyl)aminomethane, pyrogallol, ortophenantroline, L929 murine fibroblasts, Dulbecco’s Modified Eagle Medium (DMEM), fetal bovine serum (FBS), resazurin, l-glutamine, penicillin, streptomycin, and all other cell culture reagents were purchased from Sigma Aldrich (Darmstad, Germany). Glycerol, ammonia water, hydrochloric acid, sodium hydroxide, chloroform, ethyl acetate, ethanol and methanol were supplied from Chempur (Piekary Śląskie, Poland). Cell culture plasticware was purchased from VWR International (Radnor, PA, USA). All chemicals were of analytical grade.

### 2.2. Isolation, Purification and Preparation of Melanin Powder

Fresh Crimson Sweet watermelons (*Citrullus lanatus*) were purchased at a local market (Szczecin, Poland). Melanin isolation and purification were performed as described previously [10]. Briefly, watermelon seeds were first manually removed, then rinsed three times with distilled water, and finally dried at room temperature. Then, melanin was extracted by soaking 5 g of seeds in 50 mL of 1 M NaOH on an orbital shaker (150 rpm, 50 °C, 24 h), followed by centrifugation (6000× *g* rpm, 10 min) to remove plant tissue. Next, in order to precipitate the melanin, 1 M HCl was added to the alkaline mixture until the pH was 2.0, followed by centrifugation (6000× *g* rpm, 10 min). Then, the resultant pellet was first hydrolyzed in 6 M HCl (90 °C, 2 h), centrifuged (6000× *g* rpm, 10 min), and washed with distilled water five times to remove acid. After this procedure, in order to remove lipids and other residues, the pellet was washed with chloroform, ethyl acetate, and ethanol three times. Thus obtained, the purified melanin was dried and ground to a fine powder in a mortar.

### 2.3. Preparation of WPC and WPI Films

WPC/WPI-based films were prepared based on the methodology of Catarino et al. with minor modifications [24]. Briefly, film-forming solutions with a protein concentration of 10% (*w*/*w*) WPC or WPI were prepared in distilled water, at room temperature under continuous stirring. Once completely dissolved, ammonia water was added to adjust the pH to 8.0. Next, melanin was added to obtain concentrations of 0.1% and 0.5% (*w*/*w*) and stirred (250 rpm) for 1 h, until the melanin was completely dissolved. This mixture was then heated for 10 min in a water bath at 90 °C, until a uniform appearance was observed. Next, the mixture was cooled to room temperature and 5% (*w*/*w*) of glycerol (on a film-forming solution basis) was added, followed by homogenization. As reference materials, neat WPC/WPI films, without melanin addition, were also produced following the same procedure. All film samples were prepared in 10 repetitions. The film-forming solutions were cast on square (120 mm × 120 mm) polystyrene plates and dried at 40 °C for 48 h. Then, the dry films were carefully peeled off of the plates and conditioned at 25 °C and 50% RH in the clean room, prior to any tests.

### 2.4. Determination of Moisture Content, Water Solubility and Swelling Ratio

The moisture content (MC), water solubility (WS), and swelling ratio (SR) of obtained films were analyzed following the methodology of Roy et al. [14]. In brief, MC was determined as the weight change of the films after drying at 105 °C for 24 h. To determine the water solubility (WS), film specimens (2.5 cm × 2.5 cm) were first dried at 60 °C overnight and then weighed. The dried films were then dipped in 30 mL of distilled water for 24 h with occasional shaking at 25 °C, then carefully removed with a tweezer, and dried at 105 °C for 24 h, and finally re-weighed. The WS of the films was then calculated using the following formula:

(1) $$WS\;\left(\%\right)\;=\;\frac{W_{1}-W_{2}}{W_{1}}\times 100$$

where *W*_1_ is the initial and *W*_2_ is the final weight of the films, respectively.

To determine the SR of the films, pre-weighed samples were submerged in 30 mL of distilled water for 1 h. Then, surface water was carefully removed using filter paper and the samples were re-weighed. The following formula was used to calculate SR:

(2) $$SR\;\left(\%\right)\;=\;\frac{W_{2}-W_{1}}{W_{1}}\times 100$$

where *W*_1_ is initial and *W*_2_ is the final weight of the films, respectively.

### 2.5. Thickness, Mechanical, and Thermal Properties of WPC/WPI Films

The thickness of all obtained films was measured using a hand-held micrometer (Dial Thickness Gauge 7301, Mitoyuto Corporation, Kangagawa, Japan) with an accuracy of 0.001 mm. Each film was measured in five random points and the results were averaged.

The mechanical properties of the obtained films were tested using a Zwick/Roell 2,5 Z universal testing machine (Ulm, Germany). Static tensile testing was carried out to assess tensile strength and elongation at break (The gap between tensile clamps was 25 mm and crosshead speed was 100 mm/min).

Differential scanning calorimetry (DSC) measurements to assess thermal properties were carried out using a DSC calorimeter (DSC 3, Mettler-Toledo LLC, Columbus, OH, USA) over a temperature range from 30 to 300 at *φ* = 10°/min and under nitrogen flow (50 mL/min), performing two heating and one cooling scans.

### 2.6. The Water Vapour Transmission Rate (WVTR) of the Films

A gravimetric method was used to determine the Water Vapour Transmission Rate (WVTR) of the obtained films, as described previously [18]. This method relies on the sorption of humidity by calcium chloride. Briefly, 9 g of dry CaCl_2_ was placed inside a container and sealed with 8.9 cm^2^ samples of each film. Over the course of four days, the containers were weighed daily and the increase in mass indicated that water vapor passed through the films. For each film type, 10 film samples were tested, and average values for each day were calculated and used to express WVTR in g/(m^2^ × day).

### 2.7. The Water Contact Angle (WCA)

The water contact angle of all obtained films was measured using a Haas μL goniometer (Poznań, Poland). Briefly, for each film, a microsyringe was used to deposit a drop of water on the surface. Three drops were analyzed and the contact angles were averaged.

### 2.8. Spectral Analysis

The UV-Vis spectra (300–700 nm) of the film samples were measured using a UV-Vis Thermo Scientific Evolution 220 spectrophotometer (Waltham, MA, USA).

Infrared spectroscopy was used in order to assess the chemical composition of obtained films, as described previously [18]. Briefly, 4 cm^2^ squares of each film were placed directly on the ray-exposing stage of the ATR accessory of a Perkin Elmer Spectrum 100 FT-IR spectrometer (Waltham, MA, USA) operating in ATR mode. Spectra (64 scans) were recorded over a wavenumber range of 650–4000 cm^−1^, at a resolution of 4 cm^−1^. For analysis, spectra were baseline corrected and normalized using SPECTRUM software [18].

### 2.9. Color Analysis

The effect of melanin on the color of the films was measured using a colorimeter (CR-5, Konica Minolta, Tokyo, Japan). For each film type, five samples were analyzed, by making three measurements on both sides of each sample. The results (mean ± standard deviation) were expressed as L* (lightness), a* (red to green), and b* (yellow to blue). Additionally, ∆E (color difference) and YI (yellowness index), compared to unmodified WPC/WPI films, were also calculated as follows:

∆E = [(L_standard_ − L_sample_)^2^ + (a_standard_ − a_sample_)^2^ + (b_standard_ − b_sample_)]^0.5^ (3)

YI = 142.86b*L^−1^ (4)

### 2.10. Antioxidant Potential of the Films

#### 2.10.1. Reducing Power

The reducing power of the films was determined based on the previously described methodology [42] with our own modification. Briefly, film samples (100 mg) were placed in 1.25 mL of phosphate buffer (0.2 M, pH 6.6), followed by the addition of 1.25 mL of 1% potassium ferricyanide solution. Samples were then incubated for 20 min at 50 °C followed by the addition of 1.25 mL of trichloroacetic acid. Next, the test tubes were centrifuged at 3000× *g* rpm for 10 min and 1.25 mL of obtained supernatant was diluted with 1.25 mL of deionized water. Finally, 0.25 mL of 0.1% ferric chloride solution was added and the absorbance was measured at 700 nm.

#### 2.10.2. Free Radical Scavenging Activity

The free radical scavenging activity of WPC/WPI films was assessed towards ABTS, DPPH, superoxide (O_2_^−^), and hydroxyl (^·^OH) radicals. ABTS and DPPH tests were performed according to Bishai et al. with a slight modification [43]. For the ABTS test, 5 mL of 7 mM ABTS was mixed with 5 mL of 2.45 mM potassium persulfate to obtain the radical 2,2′-azino-bis(3-ethylbenzothiazoline)-6-sulphonic acid (ABTS^+^). After 16 h of incubation at room temperature protected from light, the solution was diluted to an absorbance maximum of 1.00 at 734 nm using water. To 25 mL of this ABTS^+^ solution, samples of each film (100 mg) were added and incubated up to 1 h at room temperature. As a control, tubes of ABTS^+^ solution were incubated under identical conditions, but without films. Finally, absorbance was measured and ABTS scavenging was calculated using the equation:

(5) $$Free\;radical\;scavenging\;activity\;\left(\%\right)=\;\frac{A_{sample}-A_{control}}{A_{sample}}\times 100,$$

where *A_sample_* is the absorbance of ABTS^·+^ solution with the film sample and *A_control_* is the absorbance of ABTS^+^ solution without sample.

To determine DPPH radical scavenging activity, 100 mg of each film was placed in 25 mL of 0.01 mM DPPH methanolic solution, incubated for 30 min at room temperature, and absorbance at 517 nm was measured. As a control, the same solution was measured but without any film samples. The DPPH radical scavenging activity was calculated using Equation (5).

Superoxide (O_2_^−^) scavenging activity was assessed using the pyrogallol oxidation inhibition assay, following the methodology of Ye et al. with some modification [44]. Briefly, 100 mg of each film was incubated for 5 min in 3 mL of 50 mmol/L (pH 8.2) Tris-HCl buffer with gentle stirring. Then, 0.3 mL of 7 mM pyrogallol solution that was preheated to 25 °C was added and the mixture was allowed to react for exactly 4 min. To terminate the reaction 1 mL of 10 mM HCl was added and the absorbance was measured at 318 nm. The O_2_^−^ scavenging rate was calculated from the formula:

(6) $$O_{2}^{-}\;inhibition\;\left(\%\right)=\;\left[1-\frac{\left(A_{1}-A_{1}^{\prime }\right)}{A_{0}}\right]\times 100,$$

where *A*_1_ is the absorbance of the mixture in the presence of the sample, *A*_1_′ is the absorbance of water instead of the reaction agent, and *A*_0_ is the absorbance without the sample.

Hydroxyl (·OH) scavenging was assessed using the method of Ye et al. [44] with some modification. Film specimens (100 mg) were placed in a mixture of 1.5 mL of 5 µM ortophenantroline solution and 2 mL of phosphate buffer (pH 7.4, 0.05 M). Then, 1 mL of 7.5 mM FeSO_4_ solution was added, followed by 1 mL of 0.1% H_2_O_2_, and, finally, distilled water was added to bring the total volume to 10 mL. The reaction solution was incubated at 37 °C for 1 h, protected from light, and the absorbance was measured at 536 nm. Ortophenatroline solution without H_2_O_2_ (replaced by 1 mL of methanol) served as a blank. The following formula was used to calculate hydroxyl scavenging:

(7) $$\cdot OH\;inhibition\;\left(\%\right)=\;\left[\frac{A_{2}-A_{1}}{A_{0}-A_{1}}\right]\times 100,$$

where *A*_0_ is the ortophenatroline solution without H_2_O_2_ addition, *A*_1_ is the absorbance without the sample, and *A*_2_ is the absorbance in the presence of the sample.

### 2.11. Evaluation of Cytotoxicity

In order to screen for potential cytotoxicity, extract tests and direct contact tests were carried out based on ISO 10993-5 using L929 murine fibroblasts [45]. Cells (passage 20–25) were maintained in complete growth medium: DMEM containing 10% FBS, 2 mM l-glutamine, 100 U/mL penicillin, and 100 µg/mL streptomycin. For each material, 8-mm discs were cut using a steel punch and sterilized using 20 min exposure to UV lamp in BSL-2 safety cabinet (Telstar Bio II Advance, Barcelona, Spain). Extracts were prepared by placing six 8-mm diameter discs of each material in a tissue culture plate and soaking in 1 mL of growth media (ratio: 3 cm^2^/mL) for 24 h at 37 °C. Medical grade PCL (CAPA6340) was used as a negative control, nitrile glove (Mercator Nitrylex Classic, Kraków, Poland) served as a positive (toxic) control, and as a sham extract, 1 mL of media was added to an empty well. In parallel, 10,000 L929 cells were seeded per well of a 96-well plate and allowed to adhere and spread for 24 h. Next, the media was aspirated and replaced with 100 µL of extract, six technical replicates per material. After a further 24 h of culture, cell viability was assessed using an inverted light microscope (Delta Optical IB-100, Mińsk Mazowiecki, Poland) and resazurin viability assay [46] using a fluorescent plate reader (Biotek Synergy HTX, Winooski, VT, USA) (excitation 540 nm, emission 590 nm).

For the direct contact assay, 30,000 L929 cells were seeded per well of a 48-well plate and allowed to adhere and spread for 24 h. Then, discs of each material (8 mm diameter) were placed directly on top of the cell monolayer (*n* = 5 discs per material). Cells were then maintained for another 24 h of culture and viability was assessed using an inverted light microscope and resazurin viability assay—without removal of discs—as described previously.

For both experiments, viability data obtained from resazurin assay was analyzed by subtracting blank signal (growth media only, no cells) from all other measurements and normalizing to sham-treated cells. Both the extract and direct contact assay were performed twice.

### 2.12. Statistical Analyses

Statistical comparisons were performed using Statistica version 10 (StatSoft Polska, Kraków, Poland). Differences between means were determined using analysis of variance (ANOVA) followed by Fisher’s LSD post-hoc testing with a significance threshold of *p* < 0.05. All measurements were carried out in at least triplicate.

## 3. Results and Discussion

### 3.1. Hydrodynamic Properties (Moisture Content, Water Solubility and Swelling Ratio)

The MC, WS, and SR of the WPC/WPI films are summarized in Table 1. The MC of neat WPC film was 27.16 ± 1.31%, whereas the MC of neat WPI film was 24.31 ± 0.51%. Both increased significantly as melanin content increased. This observation is in line with the findings of other authors who used melanins to modify biopolymer-based films, such as chitosan and agar [14,19]. In contrast, Yang et al. [21] noticed that the MC of alginate/poly(vinyl alcohol) films decreased with increased melanin content, similarly as observed by Roy et al. for the case of cellulose/melanin films [22]. According to Roy et al., a significant increase in the MC of polymer/melanin films may be the result of reduced polymer network interactions and, as a consequence, greater accessibility of free hydroxyl groups to absorb more water molecules [14,19].

In terms of WS, melanin addition resulted in a significant decrease in WS for both types of films (*p* < 0.05). The WS of WPC-0.5% and WPI-0.5% was reduced by approximately 20.87% and 13.50%, respectively, as compared to the neat WPC and WPI films (*p* < 0.05). Previously, a WS reduction for gelatin films modified with fungal melanin was reported [38]. Finally, for the case of SR, we observed that the addition of melanin markedly increased the SR of films (*p* < 0.05). Further, for the case of WPC films, they had a much higher SR than WPI films: the SR values of WPC-0.5% and WPI-0.5% were 147.58 ± 18.04% and 469.47 ± 21.69%, respectively and are lower than those reported previously for agar/melanin films [19]. Interestingly, Roy et al. observed that SR of chitosan films was improved by the reinforcement of melanin nanoparticles [14], but in contrast, SR was reported to be decreased in the case of agar/melanin nanocomposite films [19]. In biopolymer-based films, SR primarily depends on the cross-linking, porosity, and nature of the materials [14,47]. Thus, in the present case, the cross-linking density of the melanin-modified films might be higher and, consequently, the porosity and SR of the samples may have increased [14].

### 3.2. The Thickness, Mechanical and Thermal Properties

The thickness, mechanical, and thermal properties of the samples are presented in Table 2. The modification with melanin did not affect the thickness of WPC or WPI films (*p* > 0.05), probably because of the small amount of melanin used. However, in studies by other authors who used melanin particles at higher concentrations, an increase of thickness was observed, due to higher dry mass content [19]. Here, the addition of melanin significantly enhanced the mechanical strength of WPC and WPI films (*p* < 0.05). The tensile strength (TS) of the WPI films was higher than the corresponding WPC films; the highest TS was observed for sample WPI-0.5% (6.13 ± 0.41 MPa). However, the TS of the WPC/WPI films was lower than that reported for agar/melanin [19], chitosan/melanin [14] and poly(lactic acid)/melanin films [18]. We also observed that the elongation at break (EB) of the modified films decreased in comparison to the control samples (*p* < 0.05). The increase in TS and decrease of EB can be attributed to strong hydrogen bonding (H-bonding) interactions between melanin and the polymer matrices, which improves the mechanical properties of the films. This observation is in good agreement with the results of other authors [14,19,22]. It has also been reported that reinforcement with a low percentage (0.025% and 0.05%) of fungal melanin improved the mechanical properties of poly(lactic acid)/melanin composite films; however, at higher melanin concentration (0.2%), the TS of the films decreased [18]. These results clearly indicate that melanins (synthetic or natural) can improve the mechanical properties of polymer-based films, although the effect of melanin is highly dependent on the type of polymer matrix used and melanin concentration, which play pivotal roles in the inter- and intramolecular interactions between the polymer chains in the film [14,18,19].

In the present study, DCS measurements were also carried out to investigate the thermal characteristics of the films. Table 3 presents the melting temperatures (T_m_) and melting enthalpies (ΔH_m_) of the samples. Compared to the neat WPC/WPI films, the addition of melanin markedly increased the T_m_ and ΔH_m_ of the modified films. These changes may be due to different polymer-water interactions as a result of modification with melanin [14]. For example, Dong et al. reported that the addition of synthetic and natural melanin to PVOH improved the thermal stability of the PVOH films [40]. However, there are also several reports that modification with melanin did not affect the thermal stability of agar [19], cellulose [22], and poly(lactic acid) films [18]. At the same time, in those reports, melanins were used as fillers. Thus, it can be reasonably concluded that the different effects of melanins on thermal stability may be due to different melanin sources, differences in the polymer matrix, as well as different methods of preparation of the films [13].

### 3.3. Color

**Figure 1:**
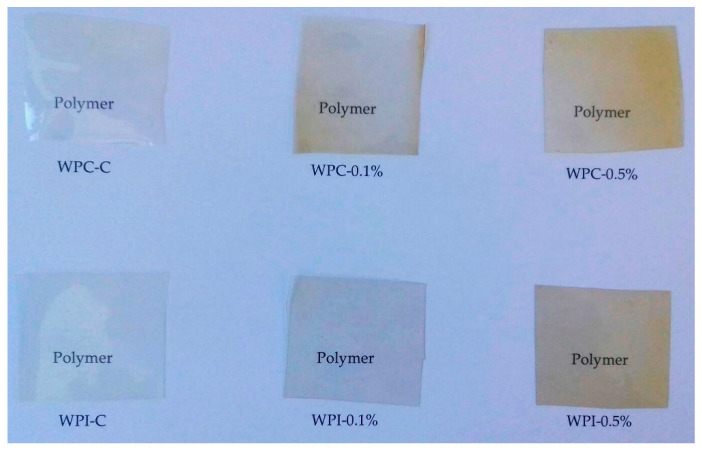
The visual appearance of neat and modified WPC/WPI films.

The apparent color, the total color difference (ΔE), and the yellowness index of the WPC/WPI films are presented in Table 3 and Figure 1. The neat WPI film was transparent and colorless, whereas neat WPC film showed a light yellowish color. Generally, WPC films were darker (lower L values) when compared with WPI films. The films with 0.1% and 0.5% melanin showed a yellowish-brown color and became darker as the concentration of melanin increased (*p* < 0.05). The redness and yellowness of the modified films also increased, due to the red-brown color of melanin. The yellowness index of WPC/WPI films increased significantly with increased melanin concentration (*p* < 0.05). As a result, the ΔE of the films was remarkably increased (*p* < 0.05) and ranged from 2.29 (WPC-0.1%) to 15.13 (WPC-0.5%). ∆E > 1 is considered perceptible to the human eye, so both concentrations of melanin in WPC/WPI films caused noticeable color changes [18]. Overall, the observed results are in good agreement with previous reports, where chitosan [14], agar [19], polypropylene/poly(butylene-co-terephthalate) [37], gelatin [38] and poly(lactic acid) [18] films were modified with various melanins.

### 3.4. UV-Barrier Properties

**Figure 2:**
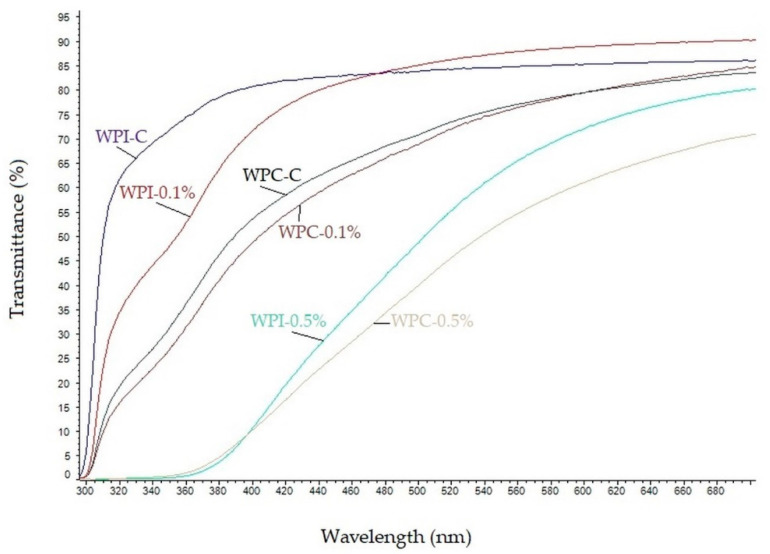
UV–Vis spectra of neat and modified WPC/WPI films.

UV-Vis light transmittance spectra of the neat and modified WPC/WPI films are shown in Figure 2. As can be seen, the neat WPC and WPI films were highly transparent to visible light at wavelengths greater than 380 nm and exhibited high transmittance for both UVA and UVB light. The transparency of WPI and WPC films decreased markedly by the addition of melanin and the decrease was concentration-dependent. These results clearly indicate that melanin, even at low concentration (0.1%) improves UV-light barrier properties of WPC, as well as WPI films. The decrease in the light transmittance of WPC and WPI films was mainly due to the absorption of UV light by melanin, similarly as in gelatin/melanin films [38]. However, when melanins were used as nanofillers, the UV barrier properties of materials were due to blocking of the light path, as was reported for poly(lactic acid)/melanin films [18] as well as for agar/melanin nanocomposite films [19]. In fact, in nature, melanins play a pivotal role in UV-protection, due to their strong UV-blocking properties resulting from the presence of phenolic and indole groups [22,37]. The high UV-barrier properties of melanin from watermelon seeds have also already been reported [10].

### 3.5. Antioxidant Activity

The antioxidant activity of the films was determined by measuring the reducing power and radical (DPPH, ABTS, O_2_^−^, OH) scavenging activity, as presented in Table 4. It was noticed that the antioxidant activity of the films was melanin concentration-dependent and increased significantly with increased melanin concentration (*p* < 0.05). The highest reducing power (1.186 ± 0.06) was measured for sample WPI-0.5%. The ABTS scavenging efficiencies of the neat WPI and WPI films were 72.02 ± 5.78% and 60.93 ± 4.50%, respectively, and an increase to 85.18 ± 1.74% and 96.87 ± 0.48%, respectively, was noticed when melanin concentration was 0.5%. A similar trend, but lower values, were observed in the case of DPPH and O_2_^−^ scavenging activity. On the other hand, the highest hydroxyl radical scavenging activity was observed for sample WPC-0.5% (39.08 ± 0.14%). In fact, the antioxidant activity of edible films is known to increase with the number of antioxidants [16,20,22,48]. Further, the antioxidant activity of the neat WPC/WPI films was expected, as they are reported to contain bioactive proteins, peptides, and amino acids with antioxidant capacity [28,31,49]. The antioxidant activity observed in the present report is also in good agreement with previous findings regarding agar [19], carrageenan [13], cellulose [22] and poly(lactic acid) [18] composite films modified with various melanins as fillers. Further, the antioxidant activity of melanin/gelatin films has been shown to increase when melanin is dissolved in a film-forming alkaline solution [38]. Likewise, it has already been observed that melanin from watermelon seeds has antioxidant activity [10]. In fact, it is well known that melanins act as effective antioxidants, because of intramolecular non-covalent electrons that can easily interact with free radicals and other reactive species [19]. Thus, the WPC/WPI melanin-modified films obtained here can be potentially used in active antioxidant packaging, to prevent oxidation of lipid-containing food or to preserve oxidation-sensitive food, as well to increase the shelf life of food [18,22,39]. Indeed, gelatin-based coatings modified with fungal melanin have been shown to have preservative activity against pork lard rancidity [39]. However, it is possible that the materials obtained here could also be used in biomedical applications, such as wound healing, due to their strong radical scavenging activity [31,50,51]. Wound healing is a complex process requiring an optimal balance between oxidative stress and antioxidant status. Typically, proper wound healing requires low levels of reactive oxygen species and low oxidative stress, while excessive oxidative stress can to impaired wound healing. As a result, antioxidants that can help control wound oxidative stress can improve wound healing [50,51].

### 3.6. FT-IR

**Figure 3:**
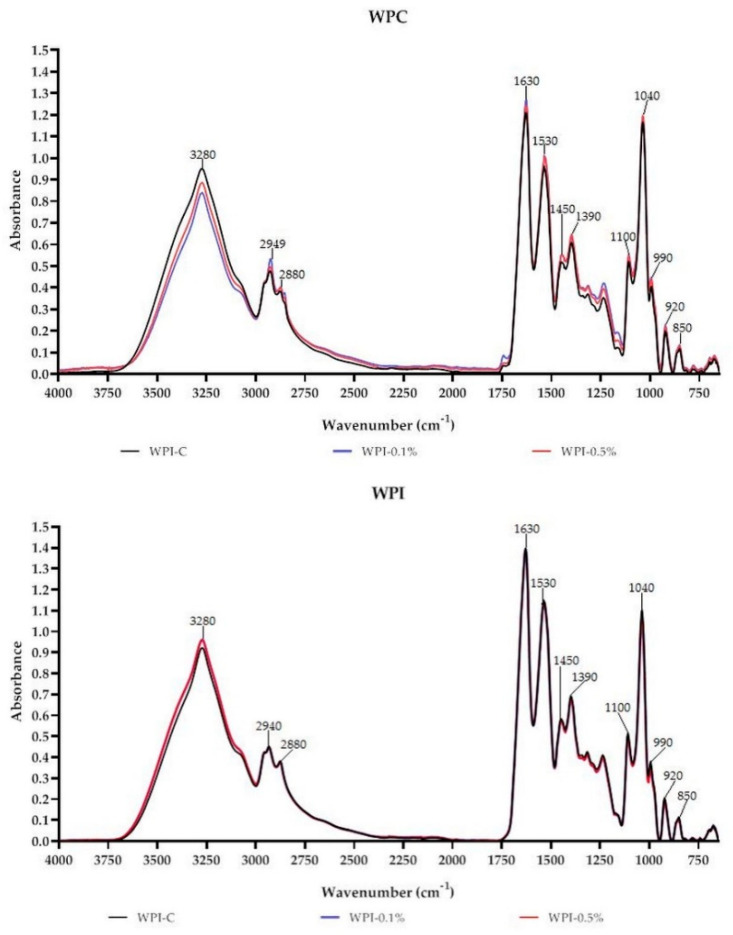
FT-IR spectra of neat WPC/WPI and melanin-modified films.

FT-IR is a commonly used technique to assess the miscibility and compatibility of biopolymers and additives, owing to its rapid and nondestructive nature [3,18,22]. Absorbance spectra of WPC-based and WPI-based films are presented in Figure 3. As can be seen the films have characteristic peaks at 3280 cm^−1^ attributed to C–H, N–H and/or O–H of WPC/WPI and melanin [10,27]. Characteristic CH_3_ and CH_2_ stretching peaks at approximately 2940 cm^−1^ and 2880 cm^−1^ were also observed. The peak centered at 1630 cm^−1^ can be attributed to Amide-I associated with C=O stretch and C–N vibrations; however, in the case of melanin-modified films, it also corresponds to the vibration of aromatic C=C of melanin [10]. Peaks in the region 1400–1550 cm^−1^ and 1200–1350 cm^−1^ can be assigned to Amide-II (groups N-H) and to Amide III (with N–H bonds and C–N stretching), respectively [27]. The region from 750 to 1200 cm^−1^ was assigned to the absorption of glycerol (plasticizer), vibrations C–O and C–C bonds [27]. In the case of WPC and WPI films modified with melanin, slight variations in peak intensities and positions were observed, particularly at 3280 cm^−1^, 2940 cm^−1^, 2880 cm^−1^ and 1630 cm^−1^. Overall, no substantial variations were noted in the functional groups of WPC/WPI modified films. The observed findings suggest that there were no structural changes in WPC and WPI films due to the addition of melanin. The small changes in intensities and minor shifts in the peaks are likely due to physical interactions (H-bonding, van der Waals force) between WPC/WPI and melanin, which is consistent with the findings of other authors [14,19,21].

### 3.7. Water Contact Angle and WVTR

The results of water contact angle (WCA) measurements are presented in Table 5. The WCA of neat WPC and WPI films was 27.67 ± 0.47° and 45 ± 0.00°, respectively. Generally, biopolymer films with a WCA of less than 65° are considered hydrophilic [14]. Concerning the effect of melanin addition, it was interestingly observed that the WCA of the films decreased significantly with increasing melanin content to 14.33 ± 0.47° and 31 ± 0.00° for samples WPC-0.5% and WPI-0.5%, respectively. This observation is in contrast to the other reports that melanins generally increase the WCA of polymeric films due to their hydrophobic nature [14,18]. However, the difference might be attributed to the use of melanin particles in those studies, whereas in the present study melanin was used to modify the alkaline film-forming solution, which could result in other interactions in the polymer matrix.

The water vapor permeability of the samples is also displayed in Table 5. It was observed that in the case of WPC, as well as WPI, the WVTR decreased as a result of modification with melanin (*p* < 0.05). The observed results are in good agreement with previous reports, where various melanins were used for the modification of alginate/poly(vinyl alcohol) [21], cellulose [22] and gelatin [38] films. However, there are also reports that the effect of melanin on WVTR depends on the concentration used. For instance, in the case of melanin added to poly(lactic acid) composite films, it was observed that WVTR decreased in the presence of low melanin content, but increased at high melanin content [18]. Likewise, in the case of agar/melanin nanoparticles composite films, the incorporation of low melanin-particle content did not result in WVTR changes, but at higher content, the WVTR increased. Whey protein-based materials are generally permeable or semipermeable to moisture penetration [25,52]. Although the WVTR of the melanin-modified films prepared here decreased (WVTR of WPC-0.5% and WPI-0.5%, 1483.53 ± 5.49 and 1490.49 ± 5.37 (g/(m^2^ × Day)) respectively), as compared to unmodified samples (WPC-C and WPI-C -1712.64 ± 7.46 and 1618.57 ± 6.23 (g/(m^2^ × Day)), respectively), they still exhibited weak water vapor barrier properties, lower than those reported in other studies [14,18,19,37].

### 3.8. Cytotoxicity

**Figure 4:**
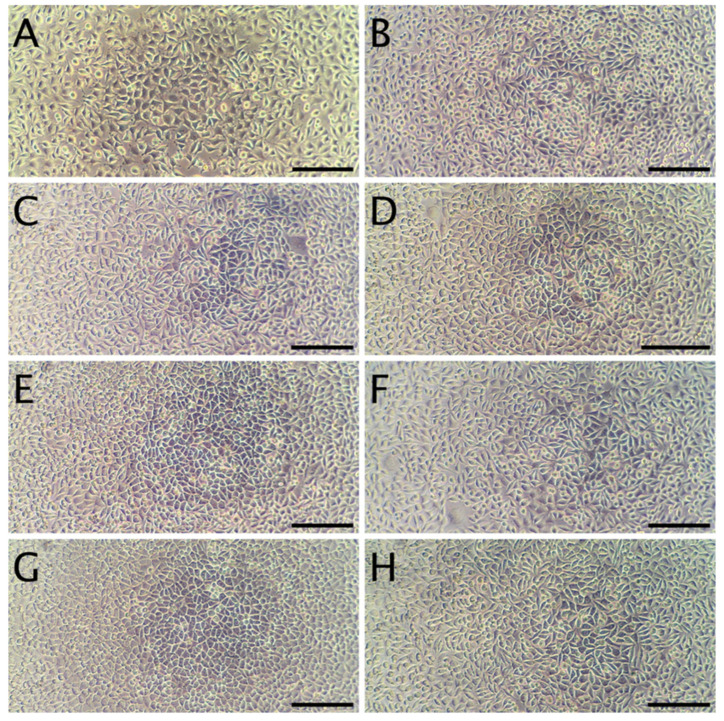
Representative micrographs of L929 fibroblasts. Panel (**A**): cells 24 h after seeding, but prior to the addition of extracts. Panel (**B**): Cells incubated for 24 h with the sham extract. Panels (**C**,**E**,**G**) present cells incubated with WPC extracts (WPC; WPC-0.1% and WPC-0.5%) for 24 h. Panels (**D**,**F**,**H**) present cells incubated with WPI extracts (WPI; WPI-0.1% and WPI-0.5%) for 24 h. The scale bar represents 200 µm.

**Figure 5:**
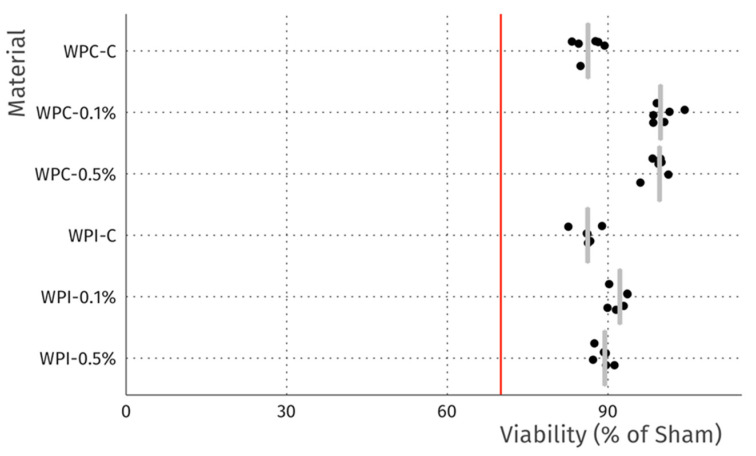
Viability of L929 fibroblasts after 24 h incubation with extracts. Data is normalized to sham extract. Dots represent technical replicates, grey bars represent median, and the red line indicates 70% viability, the threshold for cytotoxicity.

The cytotoxicity studies confirmed the non-toxic nature of the samples. As can be seen in Figure 4, after 24 h of exposure to extracts, robust cell growth was observed for all tested materials, similar to sham. Further, no marked differences in morphology were observed that could indicate a negative effect. The results of the resazurin cell viability assay, presented in Figure 5, were in good agreement with the microscopic observations. In all cases, cell viability was similar, near 90%—higher than the 70% threshold for cytotoxicity specified by the ISO 10993 norm.

**Figure 6:**
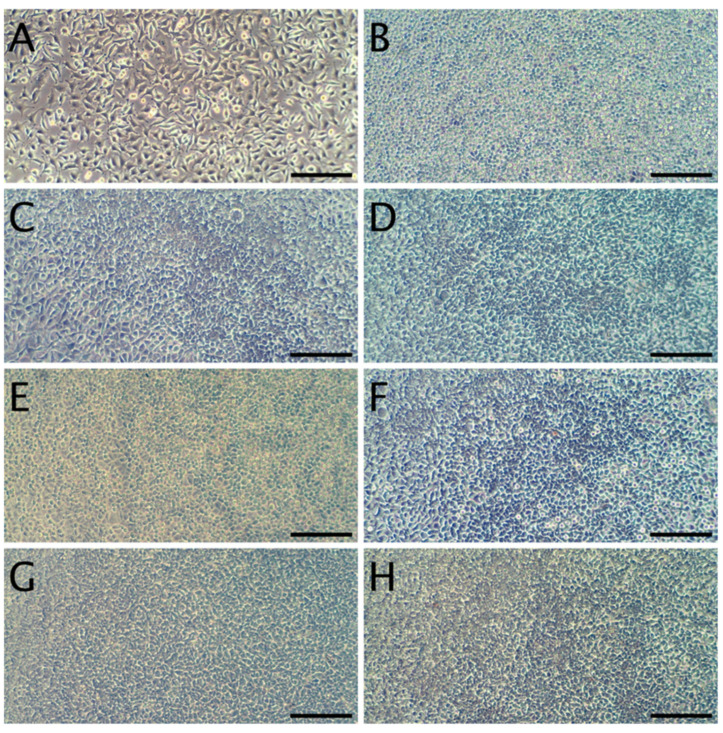
Representative micrographs of L929 fibroblasts. Panel (**A**): Cells 24 h after seeding, but prior to the addition of discs. Panel (**B**): cells incubated for 24 h without disc. Panels (**C**,**E**,**G**) present cells beneath WPC discs (WPC; WPC-0.1% and WPC-0.5%) after 24 h of culture. Panels (**D**,**F**,**H**) present cells beneath WPI discs (WPI; WPI-0.1% and WPI-0.5%) after 24 h of culture. The scale bar represents 200 µm.

**Figure 7:**
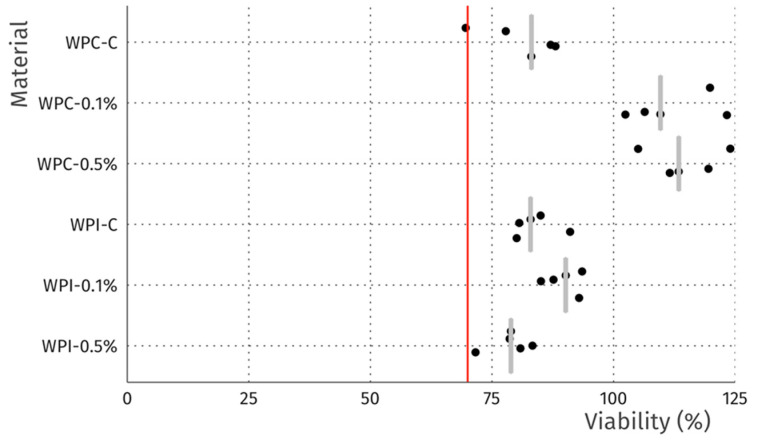
Viability of L929 fibroblasts after 24 h incubation in direct contact with discs. Data is normalized to wells without discs. Dots represent individual discs, grey bars represent median, and the red line indicates 70% viability, the threshold for cytotoxicity.

In terms of the direct contact assay, again robust cell growth was observed beneath the discs of each material, visually similar to wells without discs (Figure 6). Likewise, no marked differences in morphology were observed that could indicate a negative effect. Thus, the reactivity is graded as 0, according to ISO 10993. The resazurin viability assay indicated a modest reduction in viability for 4 of the 6 tested materials (Figure 7), which is likely due to mechanical damage to the monolayer caused by the physical presence of the disc on top of the cells. Meanwhile, the values above 100% may be due to an interaction between melanin and the resazurin reagent, as the assay was performed with the discs remaining in place to avoid the risk of further mechanical damage. To compare the viability data to the 70% viability threshold set by the ISO10993 standard, we fitted a linear model (estimated using ordinary least squares, using R (RStudio)) to predict (Normalized Viability (%)-Threshold (70%)) with Material. The model explains a significant and substantial proportion of variance (R^2^ = 0.96, F (6, 24) = 94.64, *p* < 0.01, adj. R^2^ = 0.95). For each material, the effect was positive, indicating viability values were above the threshold, and significant (*p* < 0.001). Both cell culture experiments were repeated and similar results were observed. Overall, the non-toxic nature of the films was expected, as no cytotoxic effects of whey proteins [29] nor melanins [14,53] have been reported.

## 4. Conclusions

This article explored the properties of WPC and WPI films modified with plant melanin derived from agro-industrial by-product: watermelon seeds. The properties of melanin-modified WPC/WPI films were compared to neat WPC and WPI films. The modification with melanin had a marked effect on the mechanical, antioxidant, hydrodynamic and barrier properties, but did not introduce cytotoxicity. We conclude that the obtained biofunctional WPC/WPI melanin-modified films with improved mechanical, UV-barrier, and water vapor barrier properties, along with strong antioxidant activity could be used for active food packaging or, potentially, biomedical applications, such as wound dressings.

## Figures and Tables

**Table 1:** Moisture content (MC), water solubility (WS) and swelling ratio (SR) of neat whey protein concentrate/isolate films (WPC/WPI) and melanin-modified films.

Sample	MC (%)	WS (%)	SR (%)
WPC-C	27.16 ± 1.31 ^b^	65.90 ± 6.12 ^a^	324.30 ± 68.92 ^a^
WPC-0.1	27.55 ± 0.25 ^b^	55.31 ± 4.29 ^b^	331.47 ± 18.62 ^a^
WPC-0.5	31.60 ± 0.56 ^a^	45.02 ± 2.98 ^c^	469.47 ± 21.69 ^b^
WPI-C	24.31 ± 0.51 ^a^	70.57 ± 5.24 ^a^	75.85 ± 8.58 ^a^
WPI-0.1	26.78 ± 0.38 ^b^	64.25 ± 3.54 ^a,b^	111.94 ± 11.69 ^b^
WPI-0.5	27.92 ± 0.06 ^b^	57.08 ± 6.37 ^b^	147.58 ± 18.04 ^c^

Values are means ± standard deviation. Means with different lowercase are significantly different at *p* < 0.05.

**Table 2:** Thickness, mechanical, and thermal characteristics of neat WPC/WPI and melanin-modified films.

Sample	Thickness (mm)	TS (MPa)	EB (%)	T_m_ (°C)	ΔH_m_ (J/g)
WPC-C	0.18 ± 0.02 ^a^	4.11 ± 0.36 ^a^	18.32 ± 1.28 ^a^	98.17	−68.83
WPC-0.1	0.18 ± 0.04 ^a^	4.61 ± 0.45 ^b^	12.66 ± 1.28 ^b^	99.57	−62.50
WPC-0.5	0.18 ± 0.01 ^a^	4.87 ± 1.04 ^b^	11.14 ± 1.08 ^b^	106.90	−59.30
WPI-C	0.14 ± 0.02 ^a^	4.50 ± 1.02 ^a^	16.22 ± 0.62 ^a^	103.71	−162.24
WPI-0.1	0.16 ± 0.01 ^a^	5.51 ± 1.34 ^a^	14.88 ± 2.12 ^a,b^	106.35	−125.92
WPI-0.5	0.16 ± 0.04 ^a^	6.13 ± 0.41 ^b^	13.96 ± 1.08 ^b^	108.21	−96.91

Values are means ± standard deviation. Means with different lowercase are significantly different at *p* < 0.05.

**Table 3:** Color parameters (L*, a*, b*), total color difference (ΔE), and yellowness index (YI) of neat WPC/WPI and melanin-modified films.

Sample	L*	a*	b*	ΔE	YI
WPC-C	86.42 ± 0.91 ^a^	−0.51 ± 0.10 ^a^	11.74 ± 1.73 ^a^	used as standard	19.41 ± 3.08 ^a^
WPC-0.1	85.84 ± 1.18 ^a^	−0.44 ± 0.17 ^a^	13.96 ± 2.24 ^a^	2.29 ± 0.85 ^a^	23.23 ± 4.10 ^a^
WPC-0.5	80.83 ± 1.27 ^b^	0.90 ± 0.56 ^b^	25.73 ± 3.26 ^b^	15.13 ± 0.13 ^b^	45.48 ± 7.18 ^b^
WPI-C	90.57 ± 0.11 ^a^	−0.85 ± 0.07 ^a^	4.64 ± 0.47 ^a^	used as standard	7.32 ± 0.74 ^a^
WPI-0.1	89.38 ± 0.61 ^b^	−0.72 ± 0.08 ^a,b^	7.77 ± 1.22 ^b^	3.35 ± 1.53 ^a^	12.42 ± 1.87 ^b^
WPI-0.5	86.41 ± 0.64 ^c^	−0.66 ± 0.10 ^b^	16.14 ± 1.78 ^c^	12.23 ± 1.08 ^b^	26.68 ± 3.14 ^c^

Values are means ± standard deviation. Means with different lowercase are significantly different at *p* < 0.05.

**Table 4:** Reducing power (RP) and radical (DPPH, ABTS, O_2_^−^, ^·^OH) scavenging activity of neat WPC/WPI and melanin-modified films.

Sample	RP (700 nm)	DPPH (%)	ABTS (%)	O_2_^−^ (%)	·OH (%)
WPC-C	0.661 ± 0.245 ^a^	37.70 ± 1.67 ^a^	72.03 ± 5.78 ^a^	26.80 ± 1.61 ^a^	31.68 ± 3.21 ^a^
WPC-0.1	0.863 ± 0.122 ^a^	49.87 ± 0.13 ^b^	76.86 ± 6.13 ^a,b^	29.07 ± 1.17 ^a,b^	33.45 ± 0.30 ^a^
WPC-0.5	0.924 ± 0.069 ^b^	63.68 ± 2.69 ^c^	85.18 ± 1.74 ^b^	38.11 ± 0.31 ^b^	39.08 ± 0.14 ^b^
WPI-C	0.740 ± 0.192 ^a^	58.76 ± 0.10 ^a^	60.94 ± 4.50 ^a^	27.96 ± 3.45 ^a^	32.33 ± 0.69 ^a^
WPI-0.1	1.110 ± 0.111 ^b^	65.45 ± 0.20 ^b^	95.83 ± 1.52 ^b^	33.48 ± 5.78 ^b^	33.91 ± 0.27 ^b^
WPI-0.5	1.186 ± 0.060 ^b^	72.70 ± 0.43 ^c^	96.87 ± 0.48 ^b^	38.19 ± 1.85 ^c^	37.47 ± 0.05 ^c^

Values are means ± standard deviation. Means with different lowercase are significantly different at *p* < 0.05.

**Table 5:** Water Contact Angle (WCA) and Water Vapor Transmission Ratio (WVTR) of neat WPC/WPI and melanin-modified films.

Sample	WCA (°)	WVTR (g/(m^2^ × Day))
WPC-C	27.67 ± 0.47 ^a^	1712.64 ± 7.46 ^a^
WPC-0.1	18.00 ± 0.00 ^b^	1599.23 ± 5.01 ^b^
WPC-0.5	14.33 ± 0.47 ^c^	1483.53 ± 5.49 ^c^
WPI-C	45.00 ± 0.00 ^a^	1618.57 ± 6.23 ^a^
WPI-0.1	33.00 ± 0.00 ^b^	1566.70 ± 7.14 ^b^
WPI-0.5	31.00 ± 0.00 ^c^	1490.49 ± 5.37 ^b^

Values are means ± standard deviation. Means with different lowercase are significantly different at *p* < 0.05.

## References

[1] Avramescu S.M., Butean C., Popa C.V., Ortan A., Moraru I., Temocico G. (2020). Edible and functionalized films/coatings—Performances and perspectives. Coatings.

[2] Dueñas M., García-Estévez I. (2020). Agricultural and food waste: Analysis, characterization and extraction of bioactive compounds and their possible utilization. Foods.

[3] Drozłowska E., Łopusiewicz Ł., Mężyńska M., Bartkowiak A. (2020). Valorization of flaxseed oil cake residual from cold-press oil production as a material for preparation of spray-dried functional powders for food applications as emulsion stabilizers. Biomolecules.

[4] Turon X., Venus J., Arshadi M., Koutinas M., Lin C.S.K., Koutinas A. (2014). Food waste and byproduct valorization through bio-processing: Opportunities and challenges. BioResources.

[5] Maina S., Kachrimanidou V., Koutinas A. (2017). A roadmap towards a circular and sustainable bioeconomy through waste valorization. Curr. Opin. Green Sustain. Chem..

[6] Deshmukh C.D., Jain A., Tambe M.S. (2015). Phytochemical and Pharmacological profile of *Citrullus lanatus* (THUNB). Biolife.

[7] Mehra M., Pasricha V., Gupta R.K. (2015). Estimation of nutritional, phytochemical and antioxidant activity of seeds of musk melon (*Cucumis melo*) and water melon (*Citrullus lanatus*) and nutritional analysis of their respective oils. J. Pharmacogn. Phytochem..

[8] Tabiri B. (2016). Watermelon seeds as food: Nutrient composition, phytochemicals and antioxidant activity. Int. J. Nutr. Food Sci..

[9] Seidu K.T., Otutu O.L. (2016). Phytochemical composition and radical scavenging activities of watermelon (*Citrullus lanatus*) seed constituents. Croat. J. Food Sci. Technol..

[10] Łopusiewicz Ł. (2018). Antioxidant, antibacterial properties and the light barrier assessment of raw and purified melanins isolated from *Citrullus lanatus* (watermelon) seeds. Herba Pol..

[11] Glagoleva A.Y., Shoeva O.Y., Khlestkina E.K. (2020). Melanin pigment in plants: Current knowledge and future perspectives. Front. Plant Sci..

[12] Wani A.A., Sogi D.S., Singh P., Shivhare U.S. (2011). Characterization and functional properties of watermelon (*Citrullus lanatus*) seed protein isolates and salt assisted protein concentrates. Food Sci. Biotechnol..

[13] Roy S., Rhim J.W. (2019). Carrageenan-based antimicrobial bionanocomposite films incorporated with ZnO nanoparticles stabilized by melanin. Food Hydrocoll..

[14] Roy S., Van Hai L., Kim H.C., Zhai L., Kim J. (2020). Preparation and characterization of synthetic melanin-like nanoparticles reinforced chitosan nanocomposite films. Carbohydr. Polym..

[15] Soazo M., Rubiolo A.C., Verdini R.A. (2011). Effect of drying temperature and beeswax content on physical properties of whey protein emulsion films. Food Hydrocoll..

[16] Łupina K., Kowalczyk D., Zięba E., Kazimierczak W., Mężyńska M., Basiura-Cembala M., Wiącek A.E. (2019). Edible films made from blends of gelatin and polysaccharide-based emulsifiers—A comparative study. Food Hydrocoll..

[17] Szymańska M., Karakulska J., Sobolewski P., Kowalska U., Grygorcewicz B., Böttcher D., Bornscheuer U.T., Drozd R. (2020). Glycoside hydrolase (PelAh) immobilization prevents Pseudomonas aeruginosa biofilm formation on cellulose-based wound dressing. Carbohydr. Polym..

[18] Łopusiewicz Ł., Jędra F., Mizielińska M. (2018). New poly(lactic acid) active packaging composite films incorporated with fungal melanin. Polymers.

[19] Roy S., Rhim J.W. (2019). Agar-based antioxidant composite films incorporated with melanin nanoparticles. Food Hydrocoll..

[20] Moghadam M., Salami M., Mohammadian M., Khodadadi M., Emam-Djomeh Z. (2020). Development of antioxidant edible films based on mung bean protein enriched with pomegranate peel. Food Hydrocoll..

[21] Yang M., Li L., Yu S., Liu J., Shi J. (2020). High performance of alginate/polyvinyl alcohol composite film based on natural original melanin nanoparticles used as food thermal insulating and UV–vis block. Carbohydr. Polym..

[22] Roy S., Kim H.C., Kim J.W., Zhai L., Zhu Q.Y., Kim J. (2020). Incorporation of melanin nanoparticles improves UV-shielding, mechanical and antioxidant properties of cellulose nanofiber based nanocomposite films. Mater. Today Commun..

[23] Schmid M., Merzbacher S., Müller K. (2018). Time-dependent crosslinking of whey protein based films during storage. Mater. Lett..

[24] Catarino M.D., Alves-Silva J.M., Fernandes R.P., Gonçalves M.J., Salgueiro L.R., Henriques M.F., Cardoso S.M. (2017). Development and performance of whey protein active coatings with *Origanum virens* essential oils in the quality and shelf life improvement of processed meat products. Food Control.

[25] Yoshida C.M.P., Antunes A.C.B., Antunes L.J., Antunes A.J. (2003). An analysis of water vapour diffusion in whey protein films. Int. J. Food Sci. Technol..

[26] Pires A.F., Marnotes N.G., Bella A., Viegas J., Gomes D.M., Henriques M.H.F., Pereira C.J.D. (2020). Use of ultrafiltrated cow’s whey for the production of whey cheese with Kefir or probiotics. J. Sci. Food Agric..

[27] Agudelo-Cuartas C., Granda-Restrepo D., Sobral P.J.A., Castro W. (2020). Determination of mechanical properties of whey protein films during accelerated aging: Application of FTIR profiles and chemometric tools. J. Food Process Eng..

[28] Xu R., Liu N., Xu X., Kong B. (2011). Antioxidative effects of whey protein on peroxide-induced cytotoxicity. J. Dairy Sci..

[29] Owonubi S.J., Mukwevho E., Aderibigbe B.A., Revaprasadu N., Sadiku E.R. (2019). Cytotoxicity and in vitro evaluation of whey protein-based hydrogels for diabetes mellitus treatment. Int. J. Ind. Chem..

[30] Gunasekaran S., Xiao L., Ould Eleya M.M. (2006). Whey protein concentrate hydrogels as bioactive carriers. J. Appl. Polym. Sci..

[31] Kerasioti E., Stagos D., Priftis A., Aivazidis S., Tsatsakis A.M., Hayes A.W., Kouretas D. (2014). Antioxidant effects of whey protein on muscle C2C12 cells. Food Chem..

[32] Solano F. (2014). Melanins: Skin pigments and much more—Types, structural models, biological functions, and formation routes. New J. Sci..

[33] Xu C., Chen T., Li J., Jin M., Ye M. (2020). The structural analysis and its hepatoprotective activity of melanin isolated from *Lachnum* sp.. Process Biochem..

[34] Ghadge V., Kumar P., Singh S., Mathew D.E., Bhattacharya S., Nimse S.B., Shinde P.B. (2020). Natural melanin produced by the endophytic *Bacillus subtilis* 4NP-BL Associated with the Halophyte *Salicornia brachiata*. J. Agric. Food Chem..

[35] Di Mauro E., Camaggi M., Vandooren N., Bayard C., De Angelis J., Pezzella A., Baloukas B., Silverwood R., Ajji A., Pellerin C. (2019). Eumelanin for nature-inspired UV-absorption enhancement of plastics. Polym. Int..

[36] Caldas M., Santos A.C., Veiga F., Rebelo R., Reis R.L., Correlo V.M. (2020). Melanin nanoparticles as a promising tool for biomedical applications—A review. Acta Biomater..

[37] Bang Y.J., Shankar S., Rhim J.W. (2020). Preparation of polypropylene/poly (butylene adipate-co-terephthalate) composite films incorporated with melanin for prevention of greening of potatoes. Packag. Technol. Sci..

[38] Łopusiewicz Ł., Jędra F., Bartkowiak A. (2018). New active packaging films made from gelatin modified with fungal melanin. World Sci. News.

[39] Łopusiewicz Ł., Jędra F., Bartkowiak A. (2018). The application of melanin modified gelatin coatings for packaging and the oxidative stability of pork lard. World Sci. News.

[40] Dong W., Wang Y., Huang C., Xiang S., Ma P., Ni Z., Chen M. (2014). Enhanced thermal stability of poly(vinyl alcohol) in presence of melanin. J. Therm. Anal. Calorim..

[41] Kiran G.S., Jackson S.A., Priyadharsini S., Dobson A.D.W., Selvin J. (2017). Synthesis of Nm-PHB (nanomelanin-polyhydroxy butyrate) nanocomposite film and its protective effect against biofilm-forming multi drug resistant Staphylococcus aureus. Sci. Rep..

[42] Łopusiewicz Ł., Drozłowska E., Siedlecka P., Mężyńska M., Bartkowiak A., Sienkiewicz M., Zielińska-Bliźniewska H., Kwiatkowski P. (2019). Development, characterization, and bioactivity of non-dairy kefir-like fermented beverage based on flaxseed oil cake. Foods.

[43] Bishai M., De S., Adhikari B., Banerjee R. (2014). A comprehensive study on enhanced characteristics of modified polylactic acid based versatile biopolymer. Eur. Polym. J..

[44] Ye M., Wang Y., Guo G.Y., He Y.L., Lu Y., Ye Y.W., Yang Q.H., Yang P.Z. (2012). Physicochemical characteristics and antioxidant activity of arginine-modified melanin from *Lachnum* YM-346. Food Chem..

[45] ISO 10993-5 (2009). Biological Evaluation of Medical Devices—Part 5: Tests for In Vitro Cytotoxicity.

[46] Riss T.L., Moravec R.A., Niles A.L., Duellman S., Benink H.A., Worzella T.J., Minor L., Sittampalam G.S., Grossman A., Brimacombe K., Arkin M., Auld D., Austin C.P., Baell J., Bejcek B., Caaveiro J.M.M., Chung T.D.Y. (2004). Cell Viability Assays.

[47] Jayaramudu T., Varaprasad K., Kim H.C., Kafy A., Kim J.W., Kim J. (2017). Calcinated tea and cellulose composite films and its dielectric and lead adsorption properties. Carbohydr. Polym..

[48] Łupina K., Kowalczyk D., Drozłowska E. (2020). Polysaccharide/gelatin blend films as carriers of ascorbyl palmitate—A comparative study. Food Chem..

[49] Ebaid H., Salem A., Sayed A., Metwalli A. (2011). Whey protein enhances normal inflammatory responses during cutaneous wound healing in diabetic rats. Lipids Health Dis..

[50] Fitzmaurice S.D., Sivamani R.K., Isseroff R.R. (2011). Antioxidant therapies for wound healing: A clinical guide to currently commercially available products. Skin Pharmacol. Physiol..

[51] Garraud O., Hozzein W.N., Badr G. (2017). Wound healing: Time to look for intelligent, “natural” immunological approaches?. BMC Immunol..

[52] Coltelli M., Aliotta L., Gigante V., Bellusci M., Cinelli P., Bugnicourt E., Schmid M., Staebler A., Lazzeri A. (2020). Preparation and compatibilization of PBS/Whey protein isolate based blends. Molecules.

[53] Al-Tayib O.A., Elbadwi S.M., Bakhiet A.O. (2017). Cytotoxicity assay for herbal melanin derived from *Nigella sativa* seeds using in vitro cell lines. IOSR J. Humanit. Soc. Sci..

